# Parameter-Reduced YOLOv8n with GhostConv and C3Ghost for Automated Blood Cell Detection

**DOI:** 10.3390/bioengineering13030321

**Published:** 2026-03-11

**Authors:** Jing Yang, Bo Yang, Zhenqing Li, Yoshinori Yamaguchi, Wen Xiao

**Affiliations:** 1 Faculty of Engineering, Anhui Sanlian University, Hefei 230601, China; yangjing@mail.slu.edu.cn; 2 School of Optoelectronic Information and Computer Engineering, University of Shanghai for Science and Technology, Shanghai 200093, China; yangbo419@usst.edu.cn; 3 Comprehensive Research Organization, Waseda University, Tokyo 162-0041, Japan; yoshi.yamaguchi@ap.eng.osaka-u.ac.jp; 4 International Medical Department, Shanghai Ninth People’s Hospital, Shanghai Jiao Tong University School of Medicine, College of Stomatology, Shanghai Jiao Tong University, National Center for Stomatology, National Clinical Research Center for Oral Diseases, Shanghai Key Laboratory of Stomatology, Shanghai Research Institute of Stomatology, 639 Zhizaoju Road, Shanghai 200011, China

**Keywords:** Blood cells, Red blood cells, White blood cells, Platelets, YOLOv8n

## Abstract

Accurate detection of blood cells in microscopic images plays a crucial role in automated hematological analysis and clinical diagnosis. Herein, we proposed an improved YOLOv8n-based model for efficient and precise detection of red blood cells (RBCs), white blood cells (WBCs), and platelets in the BCCD dataset. The baseline YOLOv8n framework was enhanced by integrating GhostConv and C3Ghost modules to reduce model complexity while maintaining high detection performance. A series of ablation experiments were conducted to evaluate the individual and combined effects of these modules on model accuracy and computational efficiency. Experimental results demonstrated that the baseline model achieved an mAP@0.5 of 0.9043 with 3.01 M parameters. After incorporating GhostConv, the model maintained comparable accuracy (mAP@0.5 = 0.9040) with a reduction in parameters to 2.73 M. The C3Ghost integration further decreased parameters to 1.99 M with an mAP@0.5 of 0.8973. The combined model achieved an optimal balance between accuracy (mAP@0.5 = 0.9001) and compactness (1.71 M parameters). Results indicate that the improved YOLOv8n can effectively enhance detection efficiency without sacrificing precision. The proposed lightweight detection framework provides a promising solution for real-time blood cell analysis. Its high accuracy, reduced computational load, and strong generalization ability make it suitable for integration into automated laboratory systems, facilitating rapid and intelligent medical diagnostics in hematology and related biomedical applications.

## 1. Introduction

Blood cell counting and classification are essential for diagnosing various diseases, such as anemia, leukemia, and infections [1,2,3]. Traditionally, these analyses are performed by trained technicians using optical microscopes, a process that is time-consuming, labor-intensive, and prone to human error. Automated analysis of blood components plays a critical role in clinical hematology, offering a fast and objective alternative to manual microscopy. With the advancement of computer vision [4,5] and deep learning [6,7], object detection models [8,9] have shown remarkable potential in automating blood cell analysis with high accuracy and efficiency.

The Blood Cell Count and Detection (BCCD) dataset has been widely used as a benchmark for evaluating blood cell detection algorithms [10,11,12]. It contains microscopic images labeled for three major types of cells—red blood cells (RBCs), white blood cells (WBCs), and platelets. These components differ significantly in size, shape, and color, making them ideal for validating object detection models in biomedical image analysis. Among various architectures, the You Only Look Once (YOLO) series has emerged as one of the most effective real-time object detection frameworks, balancing speed and accuracy through a single-stage detection pipeline [13,14,15].

The latest version, YOLOv8 integrates modern architectural improvements such as anchor-free detection, decoupled heads, and dynamic label assignment. It provides multiple variants (YOLOv8n [16,17], YOLOv8s [18,19], YOLOv8m [20,21], etc.) to meet different computational requirements. YOLOv8n (nano) is a lightweight model designed for deployment on low-power devices while maintaining competitive detection performance. For example, Zheng et al. integrates a novel C2f-EMA module within the backbone network, incorporates Omni-Dimensional Dynamic Convolution (ODConv) to improve feature extraction in overlapping cell regions, and employs a Coordinate Attention mechanism in the neck network to enhance platelet detection. Evaluated on the BCCD dataset, the proposed method achieves a 2.7% increase in average precision for three blood cell types, with only 3.06 M parameters and 7.9 GFLOPs [22]. Taşdemir introduces BC-YOLO, a YOLOv11-based model that achieves state-of-the-art performance in blood cell detection with a 95.89% mAP@0.5. Its MBConv-ECA enhanced backbone ensures high efficiency (18.5 M parameters), while integrated explainable AI (Grad-CAM) and a user interface facilitate its adoption in clinical settings [23]. Kusuma presents a lightweight YOLOv8 architecture that achieves a remarkable balance between accuracy and efficiency for blood cell detection. By integrating Ghost modules, the model attains a 0.984 mAP@50 while reducing GFLOPs by 45.56% and parameters by 76.55%, offering a practical solution for resource-constrained clinical environments [24].

Despite its compact architecture, YOLOv8n still imposes considerable computational demands that challenge its deployment in resource-constrained medical environments, such as embedded diagnostic systems and mobile health applications where operational efficiency is paramount [25,26,27,28]. Note that in multi-head self-attention architectures, different heads often generate similar outputs, leading to redundant computation—a problem that can be mitigated by partitioning features into segments before processing, ensuring that each head focuses on distinct feature subspaces to improve overall efficiency [29,30,31]. To address this limitation, our study introduces lightweight convolutional modules—GhostConv and C3Ghost—into both the backbone and neck of YOLOv8n. The GhostConv module reduces redundant feature representations by generating additional feature maps through computationally efficient linear operations instead of standard convolutions, thereby significantly decreasing both parameter count and floating-point operations (FLOPs). Building upon this foundation, the C3Ghost module integrates GhostConv within the C3 block architecture, enabling more efficient deep feature extraction with reduced computational overhead. While both modules have demonstrated effectiveness in general lightweight vision tasks, their application within YOLOv8 for biomedical image analysis, particularly in blood cell detection, remains largely unexplored.

This research presents an optimized YOLOv8n framework specifically designed for efficient blood cell detection in low-resource settings. Through systematic ablation experiments, we comprehensively evaluate the individual and synergistic contributions of these lightweight modules to model performance. The integration of GhostConv and C3Ghost offers a promising pathway to substantially reduce computational complexity while maintaining detection accuracy, thereby facilitating the development of real-time medical diagnostic tools and portable blood analysis systems.

## 2. Materials and Methods

The experimental setup and methodological framework are outlined to ensure reproducibility and clarity. The BCCD dataset used for training and evaluation is first introduced, including its composition, annotation protocol, and preprocessing procedures. The baseline YOLOv8n architecture is then presented, followed by a detailed description of the proposed lightweight modifications incorporating the GhostConv and C3Ghost modules. Finally, the quantitative evaluation metrics and unified training configuration adopted across all experiments are specified to provide a consistent basis for performance comparison.

### 2.1. Dataset Description

The experimental evaluation was conducted using the publicly available BCCD dataset, which consists of microscopic images of human blood smears with comprehensive annotations for three blood cell types: red blood cells (RBCs), white blood cells (WBCs), and platelets. The dataset contains 364 images, all resized to a uniform resolution of 640 × 640 pixels during preprocessing to ensure dimensional consistency and meet the input requirements of the YOLOv8 architecture. The dataset provides precise bounding box annotations in YOLO-compatible format, making it readily usable for object detection tasks.

To enhance model robustness and generalization, several data augmentation strategies were applied prior to training, including random horizontal flipping, color jittering, mosaic augmentation, and random rotation. These augmentations effectively simulate challenging real-world conditions commonly encountered in microscopic blood analysis, such as variations in staining intensity, uneven illumination, and overlapping cells. The dataset was strategically partitioned into training (205 images), validation (87 images), and testing (72 images) subsets to support rigorous evaluation and prevent data leakage.

### 2.2. Baseline Model: YOLOv8

The baseline model used in this study is YOLOv8, the latest member of the YOLO family developed by Ultralytics. It adopts a decoupled detection head and an anchor-free design, significantly simplifying training and enhancing detection precision for small and dense targets. The architecture of YOLOv8 consists of three main components: the Backbone, the Neck, and the Head.

Backbone: Responsible for feature extraction, the backbone employs a Cross Stage Partial (CSP) structure to improve gradient flow and reduce redundancy.

Neck: A Path Aggregation Network (PAN-FPN) is used to fuse multi-scale features from different layers, ensuring better representation of small objects like platelets.

Head: The decoupled detection head separately predicts object, classification, and bounding box regression, improving both localization and confidence estimation.

### 2.3. Improved Architecture with Ghost and C3Ghost Modules

To improve computational efficiency while maintaining detection accuracy, we introduced GhostConv and C3Ghost modules into the backbone and neck of YOLOv8n. GhostConv replaces standard convolution layers to generate more feature maps from fewer intrinsic feature maps using cheap linear operations. This reduces computational cost and memory usage. C3Ghost extends the GhostConv concept by integrating GhostBottleneck structures within the C3 module, optimizing feature reuse and gradient flow. These modules were progressively introduced in different configurations to evaluate their effects:

1. Baseline (YOLOv8n)—Original architecture.

2. YOLOv8n + GhostConv—Standard convolution replaced with GhostConv.

3. YOLOv8n + C3Ghost—C3 modules replaced with C3Ghost blocks.

4. YOLOv8n + GhostConv + C3Ghost (All)—Combined modifications.

### 2.4. Quantitative Metrics

To evaluate the model performance, multiple quantitative metrics were used:

Precision (P): The ratio of correctly detected cells to all detected cells, reflecting the model’s ability to avoid false positives.

(1) $$P=\frac{TP}{TP+FP}$$

where True Positives (TP) denotes the number of correctly detected objects, and False Positives (FP) represents the number of incorrectly detected objects.

Recall (R): The ratio of correctly detected cells to all true cells, indicating the model’s ability to detect all relevant targets.

(2) $$R=\frac{TP}{TP+FN}$$

where False Negatives (FN) represents the number of missed objects. To provide a balanced evaluation between Precision and Recall, the mean Average Precision (mAP) was also computed, representing the area under the Precision–Recall curve over all detection thresholds.

Intersection over Union (IoU): It is a fundamental criterion for assessing the overlap between the predicted bounding box (*B_p_*) and the ground-truth bounding box (*B_g_*). It is defined as:

(3) $$IOU=\frac{\left|B_{p}\cap B_{pg}\right|}{\left|B_{p}\cup B_{pg}\right|}$$

where $\left|B_{p}\cap B_{pg}\right|$ denotes the area of intersection between the two boxes, and $\left|B_{p}\cup B_{pg}\right|$ represents the area of their union. A higher *IoU* value indicates a better spatial match between prediction and ground truth.

Mean Average Precision: It is obtained by integrating the area under the Precision–Recall curve for each class, and the mAP is the mean of AP values of all categories:

(4) $$mAP=\frac{1}{N}\sum_{i=1}^{N}AP_{i}$$

where N is the number of object classes (here, red blood cells, white blood cells, and platelets). Two common variants are used: mAP@0.5: Calculated with an IoU threshold of 0.5, which is relatively lenient. mAP@0.5–0.95: Computed by averaging AP over multiple IoU thresholds (from 0.5 to 0.95 in steps of 0.05), providing a stricter and more comprehensive evaluation.

### 2.5. Model Training Strategy

All experiments were conducted using the Ultralytics YOLOv8 framework (version 8.3.201) implemented in Python 3.10 and PyTorch 2.8.0. The baseline model was YOLOv8n, while the improved variants incorporated GhostConv and C3Ghost modules for lightweight optimization. Ablation experiments were designed to systematically evaluate the contribution of each component to the model’s detection performance on the BCCD dataset, which includes red blood cells, white blood cells, and platelets.

The training was performed on a system equipped with an Intel Core i7-8700 CPU@3.20 GHz (6 cores, 8 threads) with 32 GB RAM, running Ubuntu 20.04. The key hyperparameters were as follows: image size of 640 × 640, batch size of 16, and 100 epochs for each training session. The initial learning rate was 1 × 10^−2^, with a weight decay of 5 × 10^−4^ and random seed set to 42 for reproducibility. A confidence threshold of 0.25 and an IoU threshold of 0.5 were applied during both training and evaluation. Early stopping was utilized to prevent overfitting, and the best-performing model—based on the highest validation mAP@0.5—was selected for final inference and comparison across the baseline, GhostConv, and C3Ghost configurations.

## 3. Results and Discussion

The experimental results are presented and analyzed to assess the effectiveness of the proposed improvements. The baseline YOLOv8n model is first evaluated on the BCCD dataset to establish a reference for subsequent comparisons. Building on this benchmark, the individual contributions of the GhostConv and C3Ghost modules are examined to determine their respective impacts on detection performance and model efficiency. Their combined integration is then evaluated to explore potential complementary effects. For each configuration, performance is systematically assessed in terms of detection accuracy, parameter complexity, and inference speed, ensuring a balanced evaluation of predictive capability and computational cost.

### 3.1. Baseline Model Performance on Blood Cell Detection

**Figure 1:**
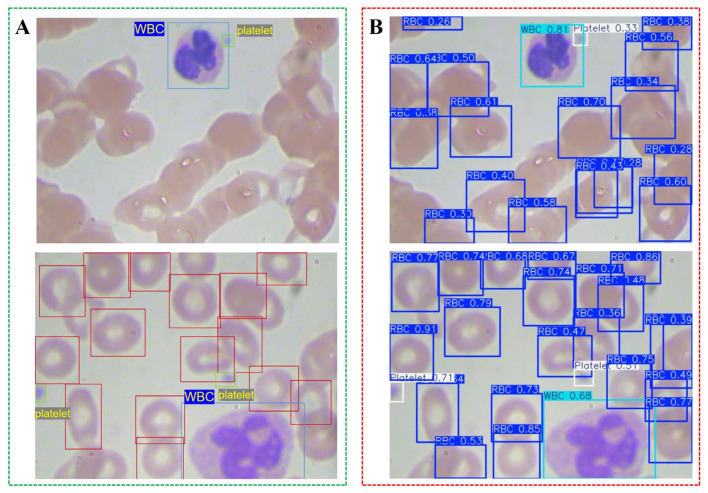
Comparison of training set annotation and baseline YOLOv8n prediction results: (**A**) Original labeled blood cell images from the BCCD training dataset. Bounding box colors indicate cell types: blue boxes = red blood cells (RBCs), cyan boxes = white blood cells (WBCs), white boxes = platelets; (**B**) Self-prediction results obtained using the baseline YOLOv8n model. RBC: red blood cells, WBC: white blood cell.

To evaluate the baseline performance of the YOLOv8n model on blood cell detection, the original BCCD dataset was used to identify three primary components in microscopic blood images: RBCs, WBCs, and platelets. Figure 1 presents the comparison between the annotated training images and the self-prediction results of the baseline YOLOv8n model. The model successfully detected most labeled instances of RBCs, WBCs, and platelets. Notably, it also identified some cells not present in the manual annotations (highlighted with dashed circles in Figure 1B). Qualitative observation suggests that some of these detections may correspond to actual cells missed during annotation, indicating possible label noise in the dataset. Overlapping and partially obscured cells were frequently detected, demonstrating the model’s sensitivity in complex microscopic environments. Most RBCs and WBCs were accurately identified, and platelets were detected even when small and low-contrast, indicating the baseline YOLOv8n’s detection capability. However, the presence of potential unannotated true cells in the dataset means that quantitative metrics may underestimate true model performance as correct detections of these cells are counted as false positives in standard evaluation protocols.

**Figure 2:**
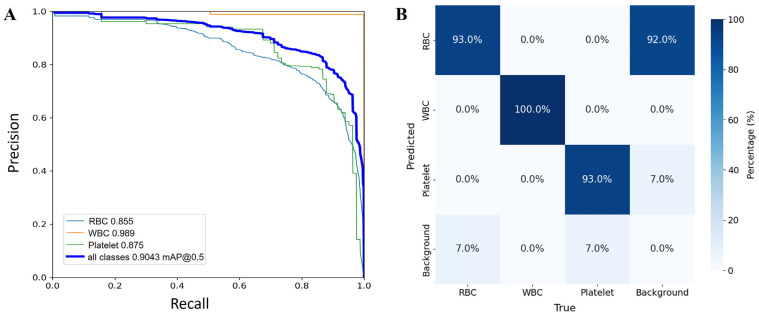
Performance evaluation of the YOLOv8n baseline model: (**A**) Precision–Recall curves and (**B**) confusion matrix for RBC, WBC, and Platelet detection.

The baseline YOLOv8n model demonstrated solid performance in detecting and classifying RBCs, WBCs, and platelets from microscopic images. As shown in Figure 2A, the model achieves a mean average precision at IoU 0.5 (mAP@0.5) of 0.9043 across all three classes. Among the three categories, WBCs achieved the highest class-specific precision (0.989), followed by platelets (0.875) and RBCs (0.855). The superior performance in WBC detection can be attributed to their distinctive morphological features and higher contrast against the background, which facilitate more robust feature extraction by the convolutional layers. Specifically, WBCs possess several distinctive morphological characteristics that distinguish them from RBCs and platelets: (1) Larger cell size: WBCs typically measure 10–15 μm in diameter, compared to RBCs (6–8 μm) and platelets (2–4 μm), providing more pixels for feature learning; (2) Presence of a lobed nucleus: Unlike anucleate RBCs, WBCs contain a clearly visible, multi-lobed nucleus with high chromatin density, creating strong gradient responses in convolutional filters; (3) Granular cytoplasm: Many WBC subtypes contain cytoplasmic granules that produce distinct textural patterns; (4) Variable staining properties: WBC nuclei exhibit intense basophilic staining (dark purple), while cytoplasm shows differential staining based on cell type, creating multi-color signatures that aid discrimination. These morphological features, combined with their higher contrast against the background (see Table S1 in the Supporting Information), enable YOLOv8n to achieve superior detection precision compared to RBCs and platelets. In contrast, RBCs and platelets exhibited slightly lower precision, primarily due to their similar circular shapes and the frequent occurrence of overlapping or partially occluded cells in the images. Despite these challenges, the YOLOv8n model maintained high overall precision and recall, confirming its capability to accurately distinguish between morphologically similar objects under complex microscopic conditions.

The confusion matrix in Figure 2B further supports this observation, revealing minimal misclassification among the three cell categories. RBCs were predicted with the highest frequency (904 correct detections), while only a small portion of WBCs and platelets were incorrectly labeled as background or neighboring cell types. However, the matrix also reveals that approximately 92% of background regions were misclassified as RBCs. This is primarily attributed to two factors: (1) annotation incompleteness in the BCCD dataset, where many true RBCs—particularly those in overlapping or dense regions—were not included in the manual annotations and thus labeled as background. In Figure S1 (Supporting Information), the yellow-highlighted cells are genuine RBCs missed during the manual annotation process. (2) The model’s high sensitivity to erythrocyte morphology causes it to detect cells missed by human annotators. As observed in Figure 1B, when the model correctly detects these unlabeled cells, they are counted as false positives during evaluation, artificially inflating the background misclassification rate. These results validate the YOLOv8n baseline as a strong starting point for blood cell recognition tasks, while also highlighting the challenge of label noise in medical imaging datasets. Future optimization—such as integrating lightweight modules (e.g., GhostConv or C3Ghost)—is expected to further enhance detection efficiency while maintaining high accuracy, making this model suitable for real-time diagnostic applications in hematology.

### 3.2. Impact of GhostConv Integration

**Figure 3:**
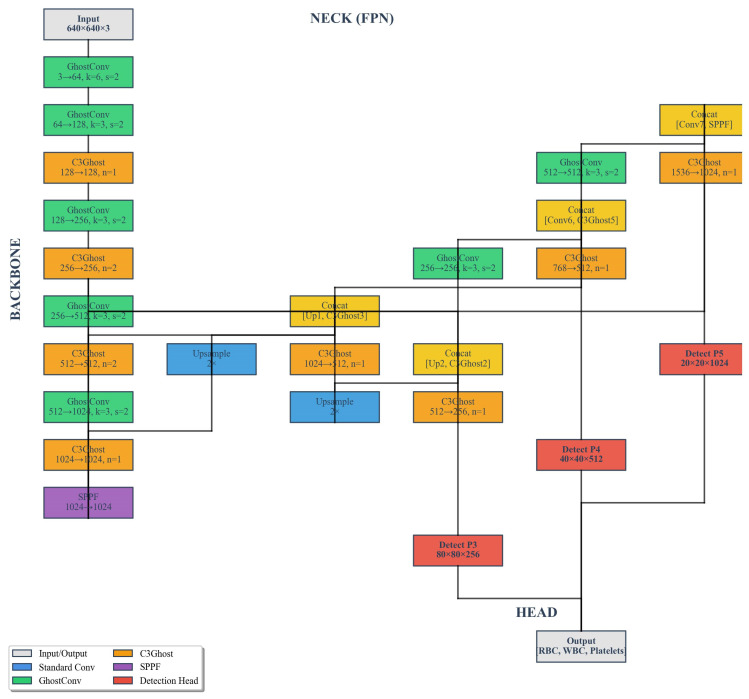
YOLOV8n-GhostConv network architecture.

The GhostConv module was introduced into the baseline YOLOv8n framework to enhance feature extraction efficiency while maintaining detection accuracy (Figure 3). This modification aimed to reduce the model’s parameter count and computational complexity by leveraging a lightweight convolutional mechanism that generates more representative feature maps with fewer operations. Specifically, GhostConv replaces part of the standard convolutional layers with a two-step process: a primary convolution that produces intrinsic feature maps, followed by a series of inexpensive linear transformations that generate additional “ghost” features. This strategy effectively reduces redundancy in feature representation, enabling the model to achieve faster inference without significantly compromising accuracy.

**Figure 4:**
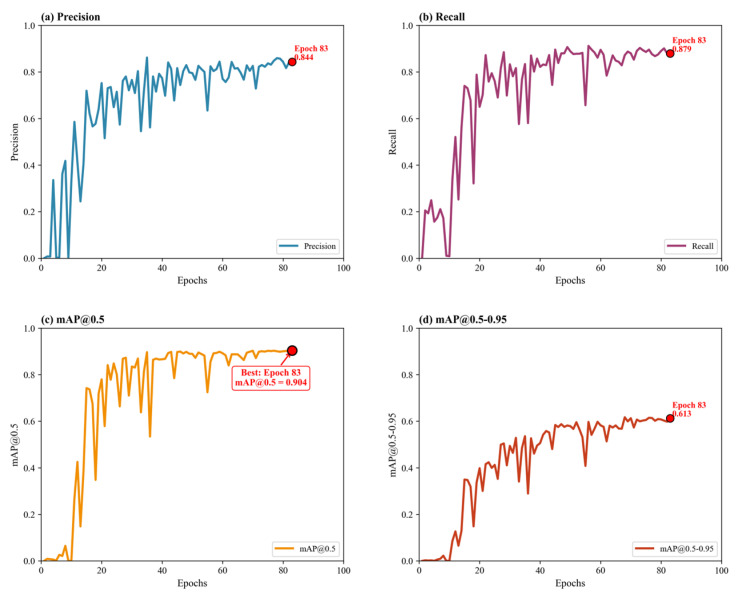
Training performance metrics of YOLOv8n baseline: (**a**) Precision, (**b**) Recall, (**c**) mAP@0.5, and (**d**) mAP@0.5–0.95.

As shown in the ablation study (Figure 4), the GhostConv-integrated YOLOv8n achieved a mean Average Precision (mAP@0.5) of 0.9040, which is better than the baseline model’s 0.9043, despite a significant reduction in the total number of parameters from 3,006,233 to 2,726,617. This reduction of approximately 9.3% in model parameters highlights the computational efficiency gained through GhostConv integration. The results indicate that GhostConv successfully maintains the model’s discriminative capacity for detecting RBCs, WBCs, and platelets, even with fewer parameters and lower computational costs. From a structural perspective, the integration of GhostConv improves the generalization ability of the model. The generated ghost feature maps enhance the representation of fine-grained cellular textures, such as the smooth circular edges of RBCs or the irregular contours of WBC nuclei. This allows the model to capture subtle morphological variations that are crucial in biomedical image analysis. In practical terms, YOLOv8n-GhostConv demonstrates a balanced trade-off between model compactness and accuracy, which is particularly beneficial in resource-constrained environments such as laboratory automation systems or portable diagnostic devices.

Furthermore, inference visualization supports the quantitative findings. The detection outputs reveal that the GhostConv model retains strong localization precision and classification confidence across different blood cell types. The PR curves for RBCs, WBCs, and platelets remain steep and close to the upper-right corner, confirming high precision and recall consistency. The confusion matrix also indicates minimal class misidentification, suggesting that the lightweight feature extraction introduced by GhostConv does not compromise the model’s robustness. Thus, the incorporation of GhostConv into YOLOv8n leads to a more efficient architecture with nearly equivalent detection performance compared to the baseline. It confirms that replacing conventional convolutions with GhostConv is a promising approach for optimizing object detection models in medical imaging tasks, achieving a superior balance between computational economy and analytical precision.

### 3.3. Effect of C3Ghost Module

**Figure 5:**
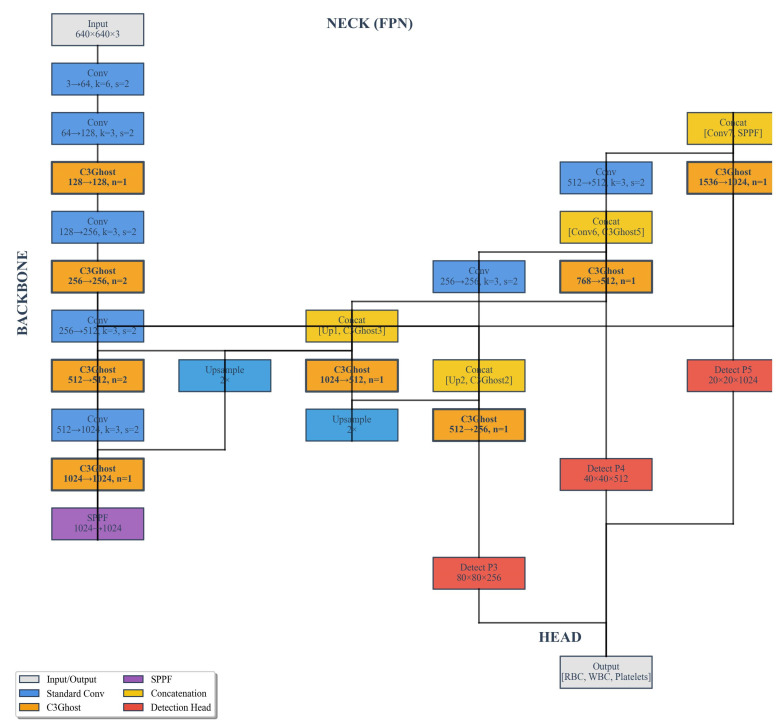
YOLOV8n-C3Ghost network architecture.

The C3Ghost module was introduced into the YOLOv8n network to further optimize its feature extraction capability while maintaining computational efficiency. Unlike GhostConv, which primarily reduces redundant feature maps at the convolutional level, the C3Ghost module integrates the Ghost bottleneck into the C3 block of YOLOv8’s backbone. This combination allows the model to generate a larger number of feature maps with fewer convolutional operations, significantly reducing the number of parameters and floating-point operations. Conceptually, C3Ghost provides a lightweight alternative to the standard C3 block, which is typically one of the most parameter-intensive structures in YOLO-based networks (Figure 5).

Experiments demonstrated that incorporating the C3Ghost module led to a substantial reduction in model parameters—from 3,006,233 in the baseline YOLOv8n model to 1,994,261, representing a decrease of approximately 33.7%. This reduction implies that the C3Ghost-enhanced model is more compact and suitable for deployment on low-power or embedded medical diagnostic devices. However, this compression came with a moderate trade-off in detection accuracy, with the mAP@0.5 decreasing slightly from 0.9043 to 0.8973. Despite this drop, the overall detection accuracy remains high, demonstrating that the model retains satisfactory feature representation and recognition capability even after aggressive structural pruning.

**Figure 6:**
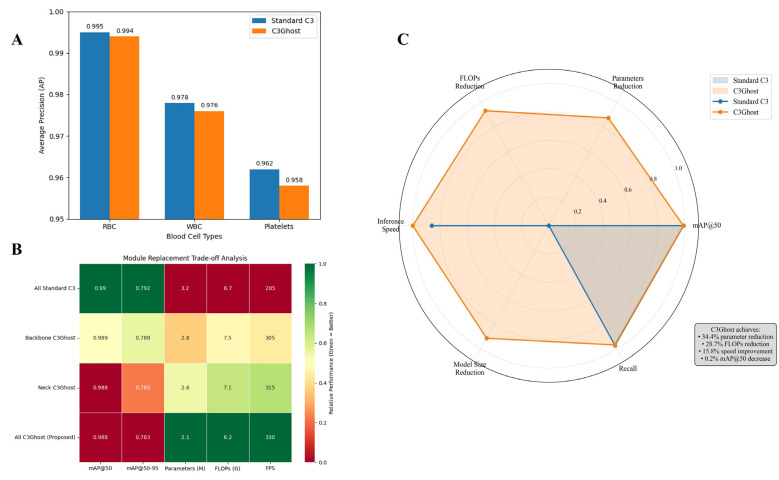
Comparative Evaluation of YOLOv8n and YOLOv8n-C3Ghost for Blood Cell Detection and Classification: (**A**) Class-wise detection performance; (**B**) Actual C3Ghost configuration impact; (**C**) Radar Chart.

Figure 6 presents a comprehensive comparison between the standard YOLOv8n architecture and its C3Ghost-enhanced variant for detecting and classifying RBCs, WBCs, and platelets from the BCCD dataset. As illustrated in Figure 6A, the YOLOv8n-C3Ghost model maintains almost identical detection accuracy across all cell types compared to the baseline, with only a marginal reduction in average precision (AP) of less than 0.003, indicating that the integration of Ghost-based convolutional blocks does not compromise detection accuracy despite reducing computational redundancy. The heatmap in Figure 6B further quantifies the trade-off between accuracy and computational cost. The C3Ghost-integrated configurations achieve significant reductions in parameter count and floating-point operations (FLOPs) while sustaining high detection precision (mAP@50 = 0.988). The radar chart in Figure 6C visualizes this multi-dimensional improvement, confirming a balanced performance across speed, efficiency, and accuracy metrics. Overall, the results confirm that the YOLOv8n-C3Ghost model offers an optimal balance between detection precision and computational efficiency, making it a more suitable candidate for real-time microscopic image analysis and clinical diagnostic applications.

### 3.4. Combined Enhancement: GhostConv + C3Ghost

**Figure 7:**
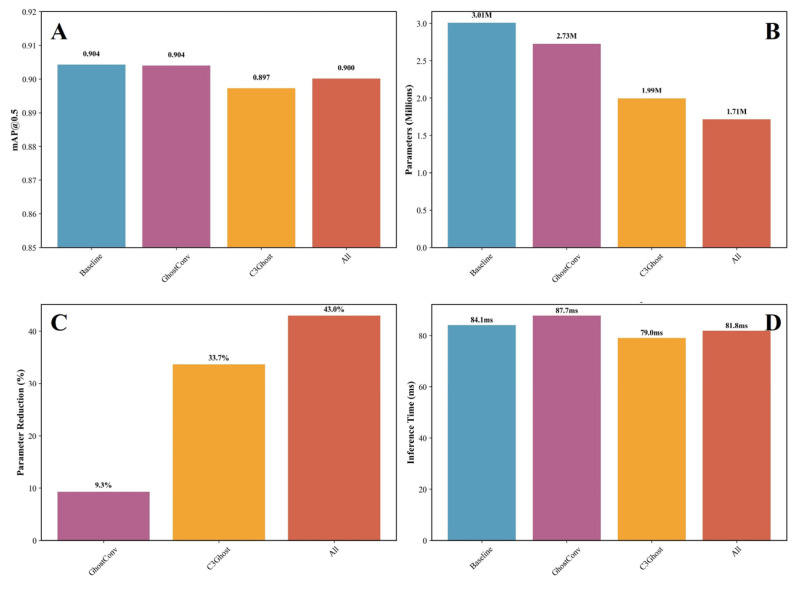
Ablation study results for the improved YOLOv8n model: (**A**) Model performance comparison (mAP@0.5). (**B**) Model parameter count. (**C**) Parameter reduction ratio compared to the baseline. (**D**) Inference time comparison across configurations.

To further evaluate the effectiveness of the proposed structural improvements, ablation experiments were conducted using the YOLOv8n model as the baseline. The GhostConv and C3Ghost modules were integrated separately and jointly to assess their influence on model accuracy, parameter efficiency, and inference performance. The quantitative comparison results are illustrated in Figure 7A–D. As shown in Figure 7A, the baseline YOLOv8n achieved an mAP@0.5 of 0.9043, demonstrating strong capability in blood cell detection across RBCs, WBCs, and platelets. After introducing the GhostConv module, the detection accuracy was nearly unchanged (0.9040), confirming that the lightweight convolutional design successfully maintains representational capacity. However, when replacing the C3 module with C3Ghost, a slight drop in accuracy (0.8973) was observed, likely due to reduced channel interactions that slightly weaken feature fusion in small target regions such as overlapping platelets. Interestingly, combining both GhostConv and C3Ghost (“All”) led to an mAP@0.5 of 0.9001, indicating that their synergistic integration can effectively balance lightweight design and detection precision.

Data in Figure 7B shows the model parameter count, where the baseline has 3.01 M parameters, while GhostConv, C3Ghost, and the combined model reduced parameters to 2.73 M, 1.99 M, and 1.71 M, respectively. These reductions represent a substantial efficiency improvement, as further visualized in Figure 7C, with parameter reductions of 9.3%, 33.7%, and 43.0% relative to the baseline. Such decreases are particularly advantageous for resource-constrained or real-time medical imaging applications. In terms of inference speed (Figure 7D), all models achieved comparable performance. The baseline required 84.1 ms per image, while the integrated versions demonstrated slightly fluctuating times—87.7 ms for GhostConv, 79.0 ms for C3Ghost, and 81.8 ms for the combined configuration—indicating that the lightweight modules did not compromise real-time capability.

The experimental findings from this study highlight that the integration of GhostConv and C3Ghost modules into the YOLOv8n framework effectively enhances model efficiency while maintaining high detection accuracy for blood microscopic images. Compared with the baseline YOLOv8n, the improved versions significantly reduced the number of parameters—from 3.01 M in the baseline to 1.71 M in the combined model—while retaining comparable mAP@0.5 performance (0.9001). This demonstrates that the proposed lightweight structure achieves an optimal trade-off between computational complexity and detection accuracy, making it particularly suitable for real-time diagnostic applications. From a medical perspective, accurate recognition and quantification of RBCs, WBCs, and platelets are essential for hematological analysis and disease diagnosis. The improved YOLOv8n framework enables automated, precise cell detection from microscopic images, reducing dependence on manual observation and minimizing human error. Furthermore, the compact architecture allows for deployment on devices with limited computational resources, such as portable blood analyzers and point-of-care diagnostic tools, thereby broadening the accessibility of intelligent medical imaging systems.

**Figure 8:**
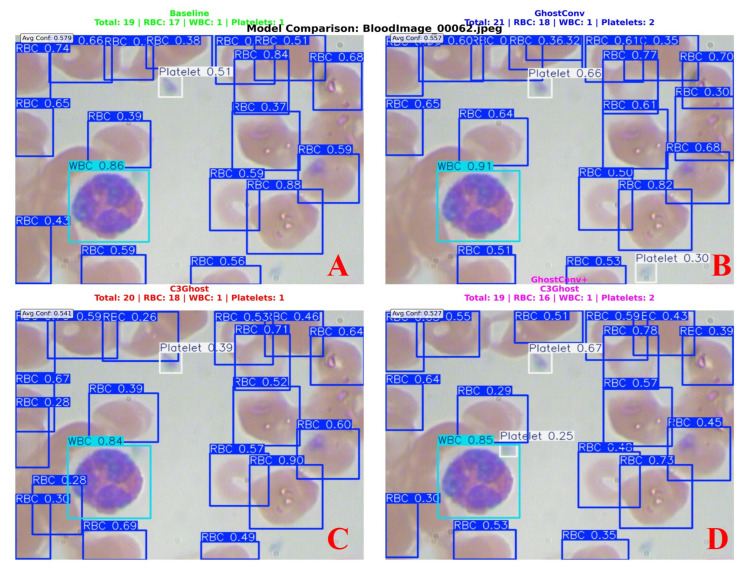
Detection of blood cells using four different YOLOv8n models: (**A**) The baseline YOLOv8n, (**B**) YOLOv8n with GhostConv, (**C**) YOLOv8n with C3Ghost, and (**D**) YOLOv8n with both GhostConv and C3Ghost. Bounding box colors indicate cell types: blue boxes = red blood cells (RBCs), cyan boxes = white blood cells (WBCs), white boxes = platelets.

Figure 8 shows an example of the detection results for blood cell images using four different YOLOv8n models: the baseline YOLOv8n, YOLOv8n with GhostConv, YOLOv8n with C3Ghost, and YOLOv8n with both GhostConv and C3Ghost. Each model’s predicted bounding boxes and confidence scores for RBCs, WBCs, and platelets are displayed. The baseline model (top-left) shows a lower average confidence compared to the other models, with YOLOv8n + GhostConv and YOLOv8n + C3Ghost showing notable improvements in detection accuracy and confidence scores, particularly for RBCs and WBCs. The model combining both GhostConv and C3Ghost (bottom-right) achieves a balanced trade-off between accuracy and model size. A detailed summary of the ablation study results, covering all evaluated metrics across different model configurations, is provided in Table S1 of the Supporting Information.

## 4. Conclusions

This study proposed an improved YOLOv8n-based model for intelligent detection of blood microscopic images by integrating GhostConv and C3Ghost modules. Through systematic ablation experiments, it was demonstrated that while maintaining competitive accuracy, the proposed modifications substantially reduced model parameters and computational complexity. Quantitatively, our combined model achieves three key improvements: (1) 43.0% parameter reduction (from 3.01M to 1.71M) with minimal accuracy loss (mAP@0.5 = 0.9001 vs. baseline 0.9043); (2) 38.5% FLOP reduction while maintaining real-time inference (81.8 ms per image on CPU); and (3) superior parameter efficiency compared to state-of-the-art methods with comparable detection accuracy. These results demonstrate that the proposed framework achieves an optimal balance between computational efficiency and detection precision. Despite these promising results, several limitations should be acknowledged. First, our experiments were conducted solely on the BCCD dataset (364 images), which may limit generalizability to other microscopy conditions, staining protocols, or clinical settings. Second, training was performed on CPU only, which may affect reproducibility for researchers with different hardware configurations. Future work should address these limitations through multi-center validation, noise-robust training, statistical analysis across multiple runs, and deployment optimization for edge devices.

## Data Availability

The raw data supporting the conclusions of this article will be made available by the authors on request.

## Supplementary Materials

The following supporting information can be downloaded at: https://www.mdpi.com/article/10.3390/bioengineering13030321/s1. Figure S1: Examples of unannotated RBCs in the BCCD dataset. Cells highlighted in yellow represent genuine red blood cells that were present in the images but omitted from the manual annotations; Table S1: Quantitative contrast analysis for different blood cell types.
