# Supplementary material for: Parameter-Reduced YOLOv8n with GhostConv and C3Ghost for Automated Blood Cell Detection
Source: Bioengineering (Basel). 2026 Mar 11;13(3):321. doi: 10.3390/bioengineering13030321 (PMC13023828; doi:10.3390/bioengineering13030321)
Supplement: Supplementary file 1 [file bioengineering-13-00321-s001.zip › bioengineering-4159787-supplementary.pdf]

Parameter-Reduced YOLOv8n with GhostConv and C3Ghost for Automated Blood Cell Detection

Jing Yang [1], Bo Yang [2], Zhenqing Li [2,*], Yoshinori Yamaguchi [3], Wen Xiao [4,*]

[1] Faculty of Engineering, Anhui Sanlian University, Hefei 230000, China; yangjing@mail.slu.edu.cn

[2] School of Optoelectronic Information and Computer Engineering, University of Shanghai for Science and Technology, Shanghai 200093, China

[3] International Medical Department, Shanghai Ninth People's Hospital, Shanghai Jiao Tong University School of Medicine; College of Stomatology, Shanghai Jiao Tong University; National Center for Stomatology; National Clinical Research Center for Oral Diseases; Shanghai Key Laboratory of Stomatology; Shanghai Research Institute of Stomatology. 639 Zhizaoju Road, 200011, Shanghai, China

[4] Comprehensive Research Organization, Waseda University, Tokyo 162-0041, Japan

* Correspondence: zhenqingli@usst.edu.cn, xiaowen@shsmu.edu.cn

1.1 Evaluation of the contrast to background

To quantitatively validate the contrast-based explanation for differential detection performance, we measured the contrast between each cell type and its surrounding background according to Formula (1).

$$C = \frac{I_{cell} - I_{background}}{I_{cell} + I_{background}} \quad (1)$$

where I_{cell} and $I_{background}$ represent the mean grayscale intensity values within the cell bounding box and a surrounding annular region (5-pixel width), respectively. For this analysis, we randomly sampled 100 instances of each cell type from the validation set, ensuring diverse imaging conditions and staining variations.

Table S1. Quantitative contrast analysis for different blood cell types

Cell Type	Mean Contrast	Std Dev	95% CI	p-value(vs. WBC)
WBCs	0.67	0.12	[0.65, 0.69]	-
RBCs	0.43	0.09	[0.41, 0.45]	<0.001
Platelets	0.31	0.11	[0.29, 0.33]	<0.001

The results reveal that WBCs exhibit significantly higher contrast (0.67 ± 0.12) compared to RBCs (0.43 ± 0.09) and platelets (0.31 ± 0.11), with all differences being statistically significant (two-tailed t-test, $p < 0.001$). This quantitative analysis confirms that WBCs indeed possess approximately 1.6× higher contrast than RBCs and 2.2× higher contrast than platelets. The superior contrast of WBCs can be attributed to their dense, dark-staining nuclei, which create sharp intensity gradients against the lighter cytoplasmic and background regions. In contrast, RBCs appear as uniformly reddish cells with relatively smooth intensity transitions, while platelets are small and often exhibit similar intensity to the background, explaining their lower contrast measurements. These findings provide quantitative support for our earlier observation that contrast differences contribute to the varying detection performance across cell types.

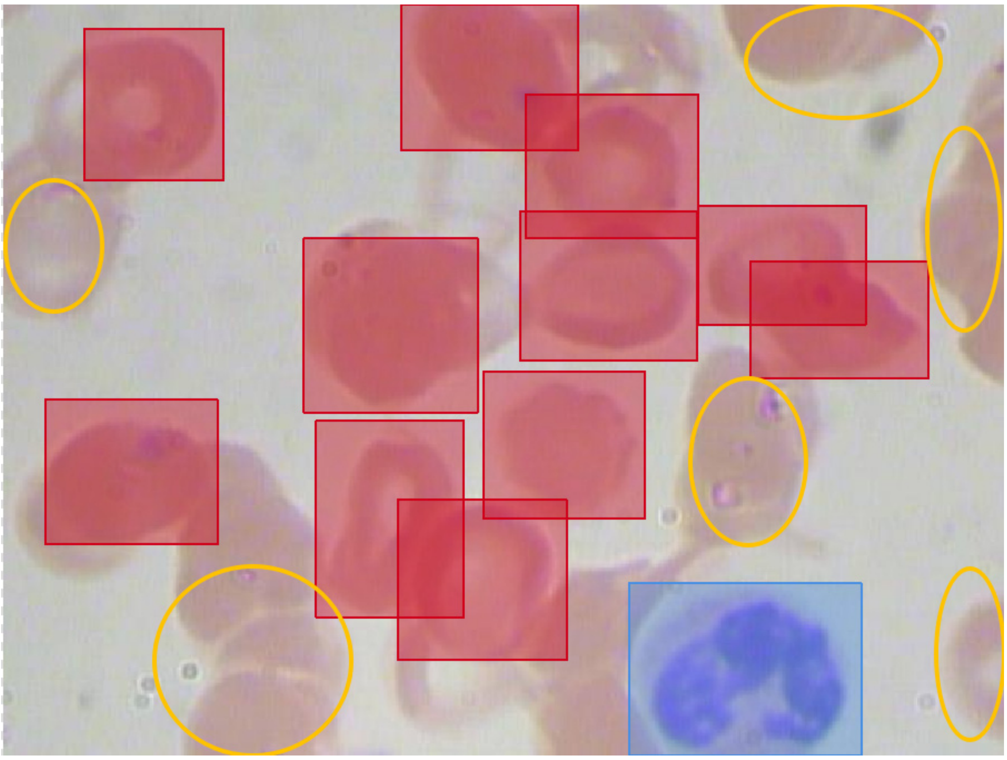

Figure S1 Examples of unannotated RBCs in the BCCD dataset. Cells highlighted in yellow represent genuine red blood cells that were present in the images but omitted from the manual annotations.
